# Metal implants influence CT scan parameters leading to increased local radiation exposure: A proposal for correction techniques

**DOI:** 10.1371/journal.pone.0221692

**Published:** 2019-08-23

**Authors:** Ok kyu Song, Yong Eun Chung, Nieun Seo, Song-Ee Baek, Jin-Young Choi, Mi-Suk Park, Myeong-Jin Kim

**Affiliations:** Department of Radiology, Yonsei University College of Medicine, Yonsei-ro, Seodaemun-gu, Korea; Northwestern University Feinberg School of Medicine, UNITED STATES

## Abstract

Metal implants not only deteriorate image quality, but also increase radiation exposure. The purpose of this study was to evaluate the effect of metal hip prosthesis on absorbed radiation dose and assess the efficacy of organ dose modulation (ODM) and metal artifact reduction (MAR) protocols on dose reduction. An anthropomorphic phantom was scanned with and without bilateral metal hip prostheses, and surface and deep level radiation doses were measured at the abdomen and pelvis. Finally, the absorbed radiation doses at pelvic and abdominal cavities in the reference, ODM, and two MAR scans (Gemstone spectral imaging, GE) were compared. The Mann Whitney-U test and Kruskal-Wallis test were performed to compare the volume CT dose index (CTDI_vol_) and mean absorbed radiation doses. Unilateral and bilateral metal hip prostheses increased CTDI_VOL_ by 14.4% and 30.5%, respectively. MAR protocols decreased absorbed radiation doses in the pelvis. MAR showed the most significant dose reduction in the deep pelvic cavity followed by ODM. However, MAR protocols increased absorbed radiation doses in the upper abdomen. ODM significantly reduced absorbed radiation in the pelvis and abdomen. In conclusion, metal hip implants increased radiation doses in abdominopelvic CT scans. MAR and ODM techniques reduced absorbed radiation dose in abdominopelvic CT scans with metal hip prostheses.

## Introduction

The number of total hip replacement surgeries with metal hip joints has gradually increased due to growing elderly populations in developed countries. In the United States, 138,700 (142.2/100,000 population) total hip replacements were performed in 2000, and this number increased to 310,800 (257.0/100,000 population) in 2010 [1]. When metal hip replacement patients undergo abdominopelvic CT scans, the metal implants produce an area of photon starvation and beam hardening, resulting in dark and bright streaks that may mask important anatomical structures or lesions in the pelvic cavity [2]. In order to minimize these metal artifacts and improve image quality, metal artifact reduction (MAR) algorithms have been developed and many studies have shown that MARs are effective in reducing metal artifacts [2–9].

In addition to creating imaging artifacts, metal implants increase radiation exposure to patients during CT scans, although it was relatively unnoticed. This is due to an increase in tube current by automated tube current modulation (ATCM) which was originally developed to reduce radiation exposure while preserving image quality [10–13]. ATCM utilizes attenuation values from scout scans to adjust tube currents based on tissue density [10]. Therefore, metal prostheses, which increase regional attenuation values in scout scans, trigger ATCM to increase tube currents near metal implants and this in turn, will cause an overall increase in radiation dose. However, increasing tube currents near metal implants offers no benefit in reducing metal artifacts [14, 15].

During the dual-energy CT acquisition for applying MAR algorithm, ATCM cannot be used. Hence, there is a possibility that we can prevent increases in radiation dose from metal implants. Furthermore, organ dose modulation (ODM) which is a recently developed radiation dose reduction technique may restrict or limit increased radiation dose caused by metal implants. ODM was developed to reduce radiation exposure to sensitive superficial organs such as the testis or breast by lowering tube currents when the X-ray tube traverses in front of the ventral aspect of the body, [16]. We hypothesized that these two techniques might reduce increases in radiation exposure caused by metal implants during CT scans. The purpose of this study is to evaluate the effect of metal hip prosthesis on absorbed radiation doses and to assess the efficacy of ODM and MAR on dose reduction.

## Materials and methods

### Phantom

A commercial anthropomorphic phantom (Model 701-G-ATOM Adult Male Phantom, CIRS, Norfolk, Virginia, USA) was used for the phantom study. Absorbed radiation doses (mGy) were measured from different parts of the phantom using the *InLight nanoDOT Dosimeter* system (*LANDAUER*, *Glenwood*, *IL*, *USA*). NanoDot dosimeters were placed in both the deep organ and surface levels of the phantom. After each scan, nanoDot dosimeters containing a single point absorbed radiation dose data were analyzed using a commercially available reader, microStar (*LANDAUER*, *Glenwood*, *IL*, *USA*).

### CT protocols

**Fig 1:**
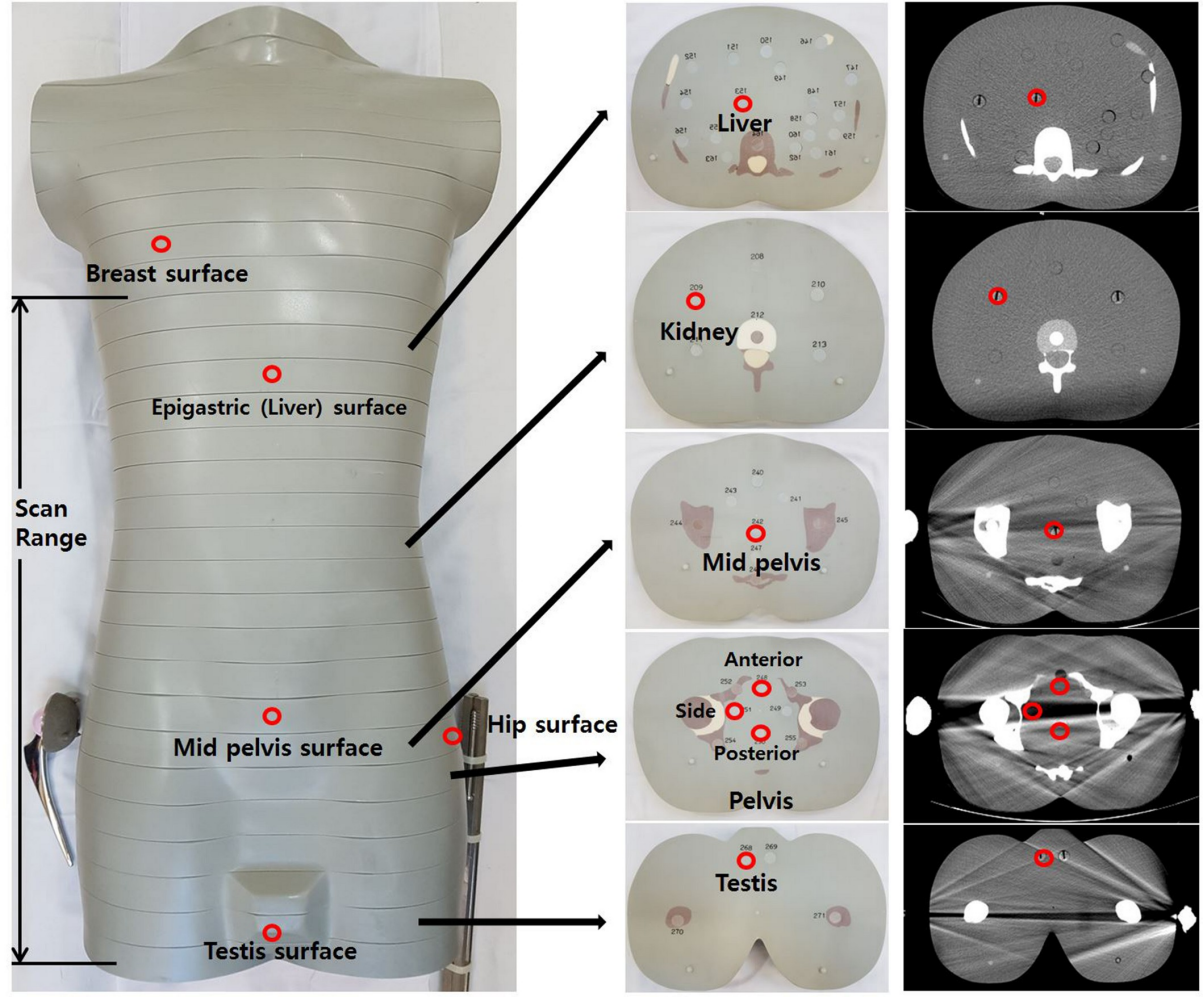
Scan range and NanoDot dosimetry locations in the deep and surface levels.

All phantom CT scans were performed with a multidetector CT *(Discovery CT 750HD*, *GE Healthcare*, *Milwaukee*, *WI*, *USA*) equipped with Gemstone technology. The dual-energy scan was performed for applying MAR, whereas all other scans were performed using single-energy. Phantoms were scanned from the upper margin of the 11^th^ thoracic vertebral body level to the upper thigh level below the testis. In order to evaluate the effect of the metal implant on radiation dose slice by slice, four CT scans were performed (phantom only, phantom with a right metal prosthesis, phantom with a left metal prosthesis, and phantom with bilateral metal prostheses) with a routine abdominopelvic CT protocol. The following CT parameters were used; rotation time, 0.5 second; detector coverage, 40 cm; tube current, less than 500 mA with ATCM; tube voltage, 120 kVp; pitch, 1.375; slice thickness, 2.5 mm; and noise index, 21.45. The metal hip prostheses and metal bars were placed next to the hip joints of the phantom to simulate actual metal hip prostheses and to create metal artifacts in the pelvic cavity (Fig 1). All scanned images were sent to the PACS and tube currents were recorded slice by slice.

In order to evaluate the effects of ODM and MAR on the absorbed radiation doses, three different types of scans were performed. First, using the routine abdominopelvic protocol, CT scans without metal (reference_no metal) and with bilateral metal hip prostheses (reference_metal) were performed to measure CTDI_VOL_ and absorbed radiation doses at designated locations in the phantom. Second, the ODM technique was applied to the reference_metal scan and the effect of ODM on the absorbed radiation dose was evaluated. ODM modulates the X-ray tube current to reduce radiation dose to the anterior aspect of the body[16]. In ODM, the scanned body is divided into anterior, both lateral, and posterior segments and the tube current is reduced when the X-ray tube traverses in front of the ventral aspect of the body. If the CTDI_VOL_ is to remain constant between the reference and ODM scans, the tube current in the dorsal segment must increase to compensate for the lowered tube current in the ventral segment. However, the ODM technique modulates the tube current in the ventral aspect of the body without increasing the tube current in the dorsal segment, thereby lowering the overall CTDI_VOL_. Last, predefined MAR scans of the phantom with bilateral metal prostheses were obtained and we evaluated the feasibility of using the MAR protocol to reduce absorbed radiation doses. The MAR algorithm with Gemstone Spectral Imaging (GSI) Dual-Energy CT mode used in this study was described in detail in a previous study [2]. In short, after acquisition of GSI dual-energy CT data, high kVp projections are used for metal segmentation, and metal-contaminated data from both high and low kVP samples are removed. Then, the iterative MAR algorithm is applied to reconstruct the missing data from forward projections in the metal segmentation domain. Preliminary images are reconstructed with estimated projections and a metal mask is added to formulate the final images. Among many predefined protocols in the console, two commonly used routine abdominal CT protocols in our institution for large-sized body (GSI3) and medium-sized body (GSI32) were selected. The following CT parameters were used: preset protocol, GSI3 (rotation time, 0.5 second; detector coverage, 40 cm; tube current, 630mA; CTDI_VOL_, 18.62 mGy) and GSI32 (rotation time, 0.5 second; detector coverage, 40 cm; tube current, 375mA; CTDI_VOL_, 13.83 mGy); fast kilovoltage switching between 80 and 140 kVp; pitch 1.375; and slice thickness, 2.5 mm. All scanned images were sent to the PACS for image review.

During all CT scans (reference_no metal, reference_metal, ODM, MAR GSI32 and GSI3 protocols), nanoDot dosimeters were placed on the surface of the phantom at breast level (not included in the scan field), mid-epigastric level, mid-pelvis, lateral hip joint, and testis to measure surface doses. Deep tissue level dosimetry locations were carefully selected and single-point absorbed radiation doses were measured in major organs such as the liver, kidney, and testis, as well as the deep pelvic cavity where photon starvation and metal artifacts mainly occur. The deep pelvis ‘side’ dosimeter location was selected to represent the area of photon starvation (Fig 1).

### Evaluation of image quality

**Fig 2:**
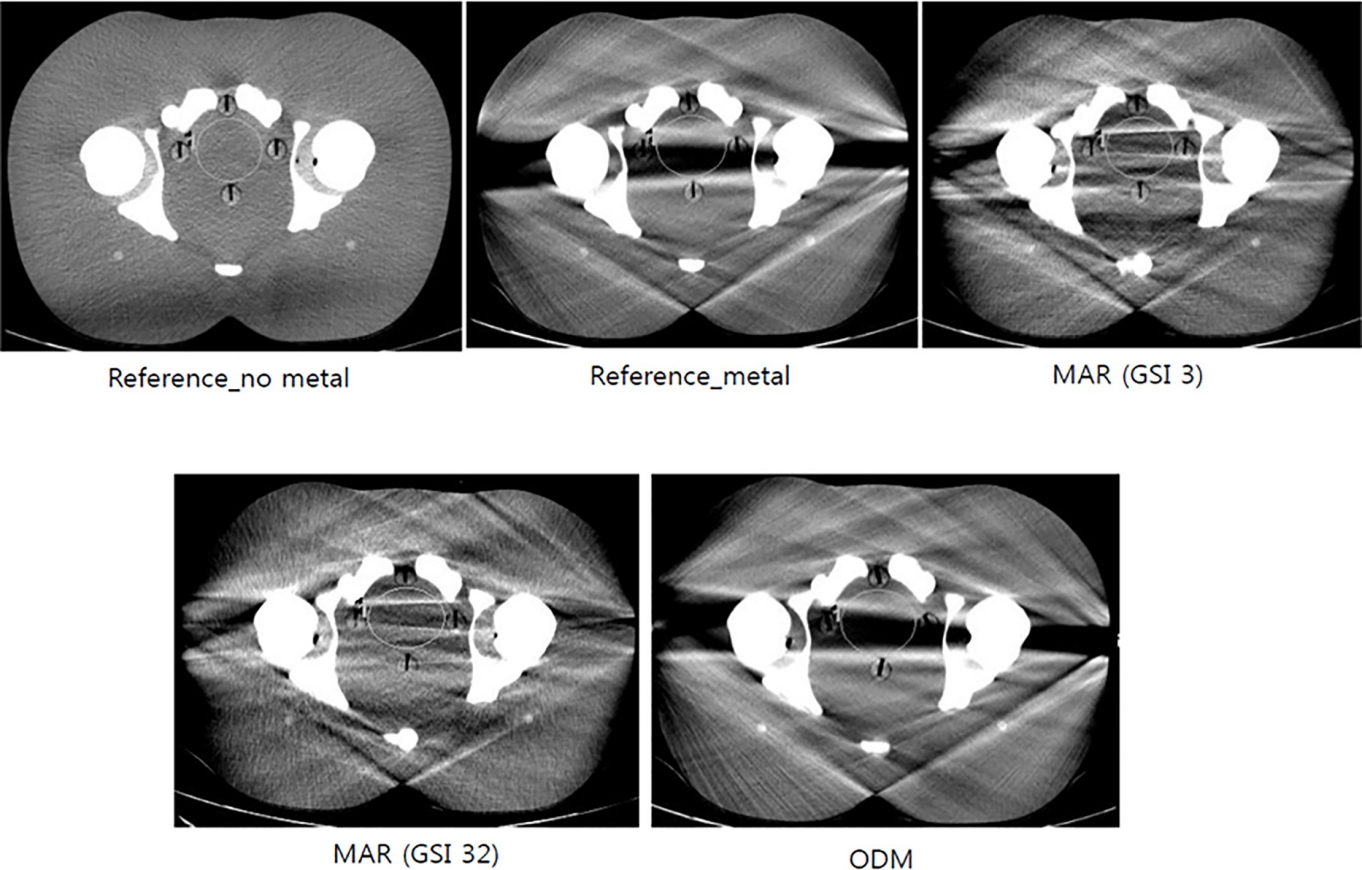
Region of Interest (ROI) for measuring the mean CT number and standard deviation (SD) of Hounsfield units (HU) in the pelvic cavity level.

In order to compare the effects of the reference, MAR, and ODM scans on metal artifacts, quantitative image analysis was performed using a PACS workstation. Region of interests (ROIs) were drawn as single circles positioned in the pelvic cavity where metal artifacts mainly occur (Fig 2). The mean CT number (HU) and standard deviations (SD) of Hounsfield units in pelvic ROIs were compared among the reference, MAR, and ODM scans.

### Statistical analysis

Each scan was repeated six times to obtain the mean and the standard deviation of the absorbed radiation. The acquired phantom data were analyzed using statistics software (SPSS, version 18.0, IBM Software). The Mann-Whitney U test was used to assess the differences in the mean absorbed radiation doses of reference scans with and without metal prostheses. The Kruskal-Wallis test with post-hoc analysis was performed to compare the mean absorbed radiation doses at different parts of the phantom, and to compare the mean CT number and the mean SD attenuation values (HU) at pelvic ROI in the reference, MAR, and ODM scans. A p-value < 0.05 was considered statistically significant.

## Results

### Effects of metal hip prosthesis on tube current and CTDI_VOL_

**Fig 3:**
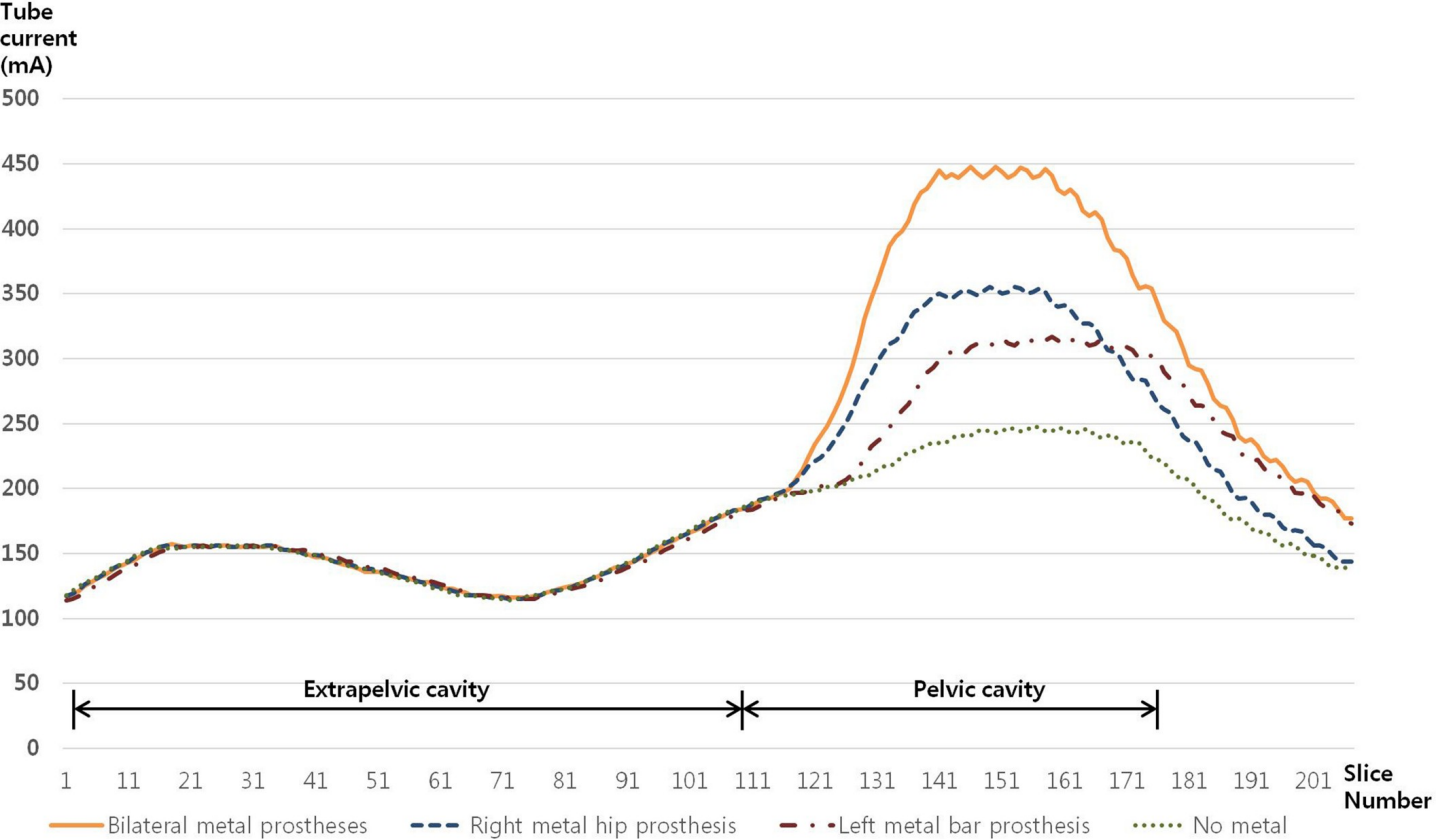
Tube currents (mAs) with and without unilateral or bilateral metal hip prostheses.

Implantation of a metal hip prosthesis increased both the tube current and CTDI_VOL_. CTDI_VOL_ in the reference_no metal scan was 13.63 mGy. When a metal prosthesis was placed on either the right or left hip joint, CTDI_VOL_ increased to 15.59 mGy (14.4%) and 15.35 mGy (12.6%), respectively. Bilateral metal prostheses increased CTDI_VOL_ to 18.14 mGy (33.1%). The tube current (mAs) also increased in the presence of a metal hip prosthesis in the pelvic cavity (Fig 3). However, metal hip prostheses had no effect on the tube currents (mA) in the mid and upper abdomen. The peak tube currents were as follows: no metal prosthesis, 247 mAs; right metal prosthesis, 355mAs; left metal prosthesis, 317mAs; and bilateral metal prosthesis, 448 mAs (Fig 3).

### Absorbed radiation dose in the reference_no metal and reference_metal scans

When metal hip prostheses were present, radiation doses absorbed in the deep and surface levels increased by 40.3 ~ 60.8% (p < 0.006) in the testis, hip, and pelvic cavity (Table 1). The pelvis side holes where photon starvation occurred showed a 50.0% increase in absorbed radiation dose (p = 0.004). Measured absorbed radiation dose at the surface level of the mid-pelvis showed the most significant increase. However, the absorbed radiation doses in abdominal organs such as the kidney and liver did not significantly increase in the presence of metal prostheses (3.6%, p = 0.200, and 2.7%, p = 0.337, respectively).

**Table 1:** Absorbed radiation dose with and without metal hip prostheses.

	Mean absorbed radiation dose (mGy) ± Standard deviation		% increase	p-value
	Reference_no metal	Reference_ metal		
Testis	20.66 ± 0.47	28.98 ± 1.50	40.3	0.004
Pelvis_mid	16.30 ± 0.29	24.95 ± 0.84	53.1	0.004
Pelvis_side	16.10 ± 0.56	24.15 ± 1.45	50.0	0.004
Pelvis_anterior	20.68 ± 0.24	32.39 ± 0.89	56.6	0.004
Pelvis_posterior	16.21 ± 0.33	25.73 ± 1.87	58.7	0.004
Kidney	16.13 ± 0.72	16.72 ± 0.67	3.6	0.200
Liver	13.04 ± 0.73	13.40 ± 0.95	2.7	0.337
Testis surface	18.01 ± 1.78	22.79 ± 1.12	26.5	0.004
Mid-pelvis surface	30.2 ± 5.08	48.86 ± 5.84	61.8	0.004
Hip surface	22.14 ± 2.63	29.46 ± 3.16	33.1	0.006
Liver surface	16.68 ± 0.92	17.70 ± 1.40	6.1	0.150
Breast surface	1.71 ± 0.05	1.87 ± 0.05	9.4	0.004

### Effect of ODM on absorbed radiation dose

**Fig 4:**
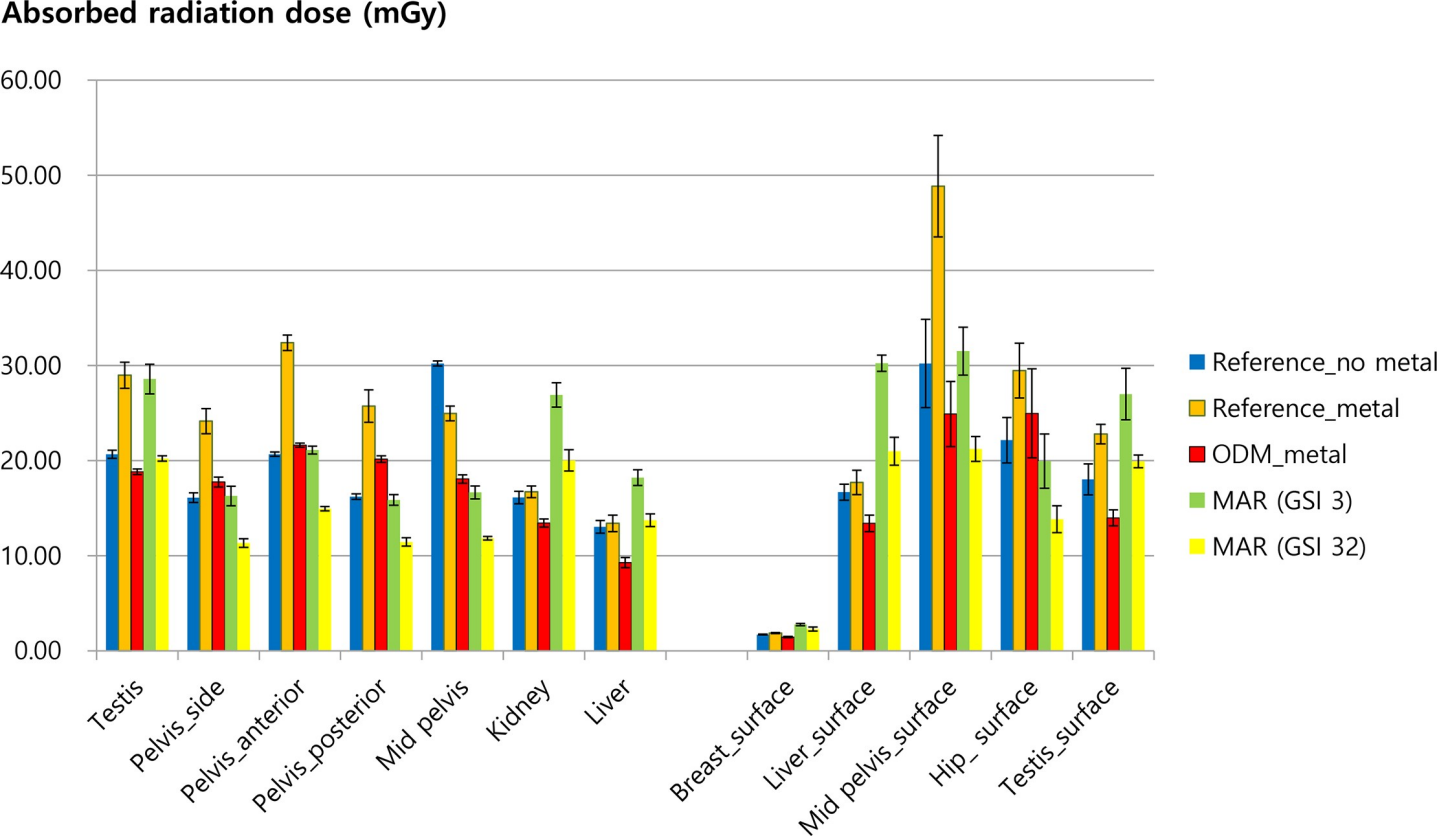
Mean absorbed radiation doses (mGy) of the deep organ and surface tissue levels in the reference_metal, ODM, and MAR scans.

When the ODM technique was applied to the reference_metal scan, the absorbed radiation dose significantly decreased in both the deep and superficial levels of the pelvic and abdominal cavities (Fig 4). Compared to the reference_metal or MAR scans, ODM showed the most significant surface dose reduction in the anterior aspect of the phantom at the liver, mid-pelvis, and testis (Fig 4, S1 Fig, and S1 Table). CTDI_VOL_ decreased from 18.14 mGy to 14.68 mGy (19.07% reduction) after applying ODM (Table 2).

**Table 2:** Volume CT dose index (CTDI_vol_) of the reference, ODM, and MAR scans.

	Reference_no metal	Reference_metal	ODM with metal	MAR (GSI3) with metal	MAR (GSI32) with metal
CTDI_VOL_ (mGy)	13.64	18.14	14.68	18.62	13.83

Each scan was repeated six times. All six scans showed constant CTDI_vol_ since the same parameters and scan fields were applied in all repeat scans.

### Effect of the MAR protocol on absorbed radiation dose

Applying the MAR protocols decreased both the surface and deep organ doses in the pelvis (Fig 4). GSI32 with CTDI_VOL_ equivalent to the reference_no metal scan showed the most significant dose reduction in the deep pelvic cavity followed by GSI3 and ODM. However, the MAR (GSI32, GSI3) protocols increased radiation doses in the upper abdominal cavity compared to the reference_metal scan. The CTDI_VOL_ of MAR scans were 18.62 mGy for GSI3 protocol and 13.83 mGy for GSI32 protocol (Table 2).

### Quantitative analysis of the mean and SD attenuation values of pelvic ROI among the reference, MAR, and ODM scans

The SD attenuation values of pelvic ROIs were significantly different among the reference_no metal, reference_metal, MAR, and ODM protocols (Table 3). The reference_no metal scan showed the lowest SD attenuation followed by MAR (GSI3 and GSI32) scans. There was no significant difference of the SD attenuation (HU) between Reference_metal and ODM_metal (p = 0.209), and between GSI3 and GSI32 (p = 1.000). The CT numbers of pelvic ROIs were significantly different among the reference_no metal, reference_metal, MAR, and ODM protocols (Table 3).

**Table 3:** Mean CT numbers (HU) and standard deviations (SD) of HU in the pelvic ROI among the reference, MAR, and ODM scans of the pelvic cavity.

	Reference_no metal	Reference_metal	MAR(GSI3)	MAR(GSI32)	ODM_metal	P
CT number (HU)^#^	33.7 ± 0.33	-46.7± 10.09	-3.3± 3.76	-3.2± 4.65	-52.8± 3.80	<0.001
SD (HU)*	20.3 ± 0.23	118.2 ± 4.66	47.9±9.68	67.4 ± 3.51	118.0 ± 8.21	<0.001

#: On post-hoc analysis, the CT numbers were not significantly different between Reference_metal and ODM_metal (p >0.999) and between GSI3 and GSI32 (p > 0.999).

*: On post-hoc analysis, there was no significant difference in the SD attenuation (HU) between GSI3 and GSI32 (p > 0.999) and between Reference_metal and ODM with metal (p = 0.209).

p: Calculated p value in the Kruskal-Wallis test.

## Discussion

Implantation of unilateral and bilateral metal hip prostheses increased the CTDI_VOL_ up to 14.4% and 33.1%, respectively. Both tube currents and absorbed radiation doses measured with dosimeters increased significantly in the pelvic cavity where metal prostheses were placed. The ODM and MAR (GSI3, GSI32) protocols effectively reduced surface and deep tissue level radiation exposure near the metal implants. MAR (GSI32) showed the most significant dose reduction in the deep pelvic cavity. ODM also showed dose reduction in both the surface and deep tissue levels of the upper abdominal cavity, whereas the MAR protocols increased absorbed radiation in the upper abdomen compared to the reference scan, possibly because ATCM was turned off during MAR scans. Consequently, there was no lowering effect on the tube current by ATCM in the metal-implant free part of the body. In addition to lowering absorbed radiation dose near metal implants, MAR improved image quality in the presence of metal prosthesis. There was no significant difference in image quality between the reference and ODM protocols.

A previous phantom study indicated that the mean CTDI increased by 48% when a metal prosthesis was present in the ATCM setting [15]. The positive oral contrast also attributed to increasing the CTDI_VOL_ approximately 6.1–11.0% compared to the neutral oral contrast agent (water) [17]. Radiation dose increases when a high attenuating material such as a metal implant or positive oral contrast is present because ATCM increases tube currents in an attempt to maintain image quality in a highly attenuating area. Our study also demonstrated similar results, with CTDI_VOL_ increasing up to 33.1% in the presence of metal hip prosthesis in an anthropomorphic phantom. The difference in the extent of radiation increase was probably caused by the different shapes, sizes, and locations of the metal prostheses in the phantom.

We anticipated that the single-point absorbed radiation dose would decrease in the area of dark streaks in the pelvic cavity because of photon starvation. However, interestingly, the absorbed radiation dose increased by 50.0% (p = 0.004) in the photon starvation area when metal hip prostheses were present. This may be caused by the increased X-ray beam density emitted from the anterior and posterior aspects. In order to compensate for increased photon attenuation caused by metal prosthesis, ATCM increases photon density by modulating tube currents.

For abdominopelvic CT scans in patients with metal hip prostheses, the MAR technique may serve as an ideal protocol. Many previous studies have documented the effectiveness of improving image quality with various MAR algorithms [2–6]. In addition to improving image quality, MAR was able to lower radiation exposure near metal implants.

Application of the ODM technique in the reference scan with bilateral metal prostheses effectively lowered both the surface and deep organ level doses. As stated earlier, ODM reduces the overall exposed radiation dose by reducing the current. Our study demonstrated that the resultant pixel noise standard deviation and CT number in ODM scans did not show statistically significant differences compared to the non-ODM scans. Therefore, the ODM technique can be utilized in metal implant patients without significantly increasing image noise.

One limitation of this study is that we only analyzed two predefined GSI protocols from a single vendor. Since we used a single CT scanner for phantom study, the results may be vendor-specific. For example, different MAR algorithms from other vendors using post-scan image processing may have different effects on CTDI_VOL_ or absorbed radiation. Secondly, it is not possible to implant metal prostheses in the phantom, and therefore, the metal prostheses were placed next to the hip joints. This may create a bias since the actual bony density still remains in the phantom, whereas in the metal hip replacement patients, bony structures in the hip joint is removed. However, we believe that this bias is not significant since the density of metal is far greater than that of bony tissue, and the metal artifact was successfully simulated in our CT scans, despite geometric discrepancies. Finally, to validate the results of our study we need data from actual patients, which may be the scope of future study.

In conclusion, metal hip implants increased radiation exposure in abdominopelvic CT scans. Metal artifact reduction and organ dose modulation techniques reduced absorbed radiation dose during abdominopelvic CT scans with metal prosthesis.

## Supporting information

S1 Fig Mean absorbed radiation doses (mGy) of the organ and surface level tissues using the reference_metal, ODM, and MAR (GSI3, GSI32) protocols. (DOCX)

S1 Table Mean absorbed radiation doses (mGy) of the deep organ and surface level tissues in the reference, ODM, and MAR (GSI3 and GSI32) scans. (DOCX)
