# Supplementary material for: Metal implants influence CT scan parameters leading to increased local radiation exposure: A proposal for correction techniques
Source: PLoS One. 2019 Aug 23;14(8):e0221692. doi: 10.1371/journal.pone.0221692 (PMC6707604; doi:10.1371/journal.pone.0221692)

S1 Fig. Mean absorbed radiation doses (mGy) of the organ and surface level tissues using the reference_metal, ODM, and MAR (GSI3, GSI32) protocols.


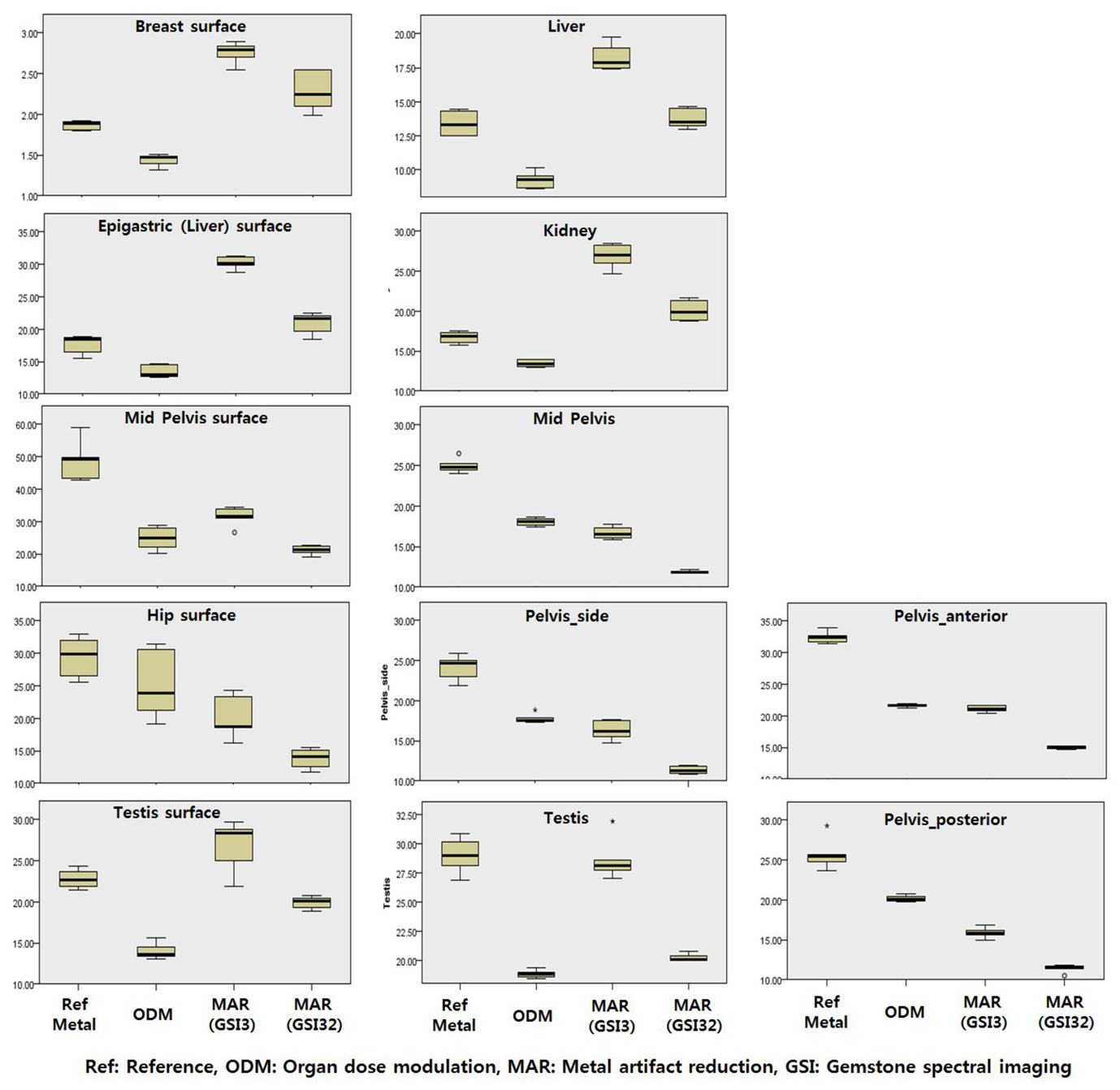

Supplement: S1 Fig — (DOCX) [file pone.0221692.s001.docx]
