# Supplementary material for: Metal implants influence CT scan parameters leading to increased local radiation exposure: A proposal for correction techniques
Source: PLoS One. 2019 Aug 23;14(8):e0221692. doi: 10.1371/journal.pone.0221692 (PMC6707604; doi:10.1371/journal.pone.0221692)
Supplement: S1 Table — (DOCX) [file pone.0221692.s002.docx]

S1 Table. Mean absorbed radiation doses (mGy) of the deep organ and surface level tissues in the reference, ODM, and MAR (GSI3 and GSI32) scans.

|  | Absorbed radiation (mGy) | | | | |  |
| --- | --- | --- | --- | --- | --- | --- |
| **Deep organ level** | Reference_no metal | Reference_ metal | ODM | MAR (GSI3) | MAR (GSI32) | p value |
| Testis | 20.66 | 28.98 | 18.82 | 28.57 | 20.22 | < 0.001 |
| Pelvis_side | 16.10 | 24.15 | 17.74 | 16.28 | 11.32 | 0.005 |
| Pelvis_anterior | 20.68 | 32.39 | 21.63 | 21.11 | 14.95 | 0.004 |
| Pelvis_posterior | 16.21 | 25.73 | 20.16 | 15.86 | 11.44 | < 0.001 |
| Mid-pelvis | 30.22 | 24.95 | 18.06 | 16.66 | 11.84 | 0.001 |
| Kidney | 16.13 | 16.72 | 13.45 | 26.91 | 20.03 | < 0.001 |
| Liver | 13.04 | 13.40 | 9.27 | 18.21 | 13.73 | 0.007 |
| **Surface Level** |  |  |  |  |  |  |
| Breast | 1.71 | 1.87 | 1.44 | 2.76 | 2.28 | < 0.001 |
| Liver | 16.68 | 17.70 | 13.40 | 30.23 | 20.98 | 0.001 |
| Mid-pelvis | 30.22 | 48.86 | 24.89 | 31.51 | 21.23 | 0.001 |
| Hip | 22.14 | 29.46 | 24.96 | 19.95 | 13.84 | 0.004 |
| Testis | 18.01 | 22.79 | 13.97 | 26.99 | 19.92 | < 0.001 |
